# Patterns, Predictors, Variations, and Temporal Trends in Emergency Medical Service Hospital Prenotification for Acute Ischemic Stroke

**DOI:** 10.1161/JAHA.112.002345

**Published:** 2012-08-24

**Authors:** Cheryl B. Lin, Eric D. Peterson, Eric E. Smith, Jeffrey L. Saver, Li Liang, Ying Xian, DaiWai M. Olson, Bimal R. Shah, Adrian F. Hernandez, Lee H. Schwamm, Gregg C. Fonarow

**Affiliations:** From Duke–National University of Singapore, Singapore (C.B.L.); Duke Clinical Research Institute, Durham, NC (E.D.P., L.L., Y.X., D.M.O., B.R.S., A.F.H.); Department of Clinical Neurosciences, Hotchkiss Brain Institute, University of Calgary, Alberta, Canada (E.E.S.); Department of Neurology, University of California, Los Angeles (J.L.S.); Division of Cardiology, University of California, Los Angeles (G.C.F.); Stroke Service, Massachusetts General Hospital, Boston (L.H.S.)

**Keywords:** stroke, emergency medicine, hospitals, registries

## Abstract

**Background—:**

Emergency medical services (EMS) hospital prenotification of an incoming stroke patient is guideline recommended as a means of increasing the timeliness with which stroke patients are evaluated and treated. Still, data are limited with regard to national use of, variations in, and temporal trends in EMS prenotification and associated predictors of its use.

**Methods and Results—:**

We examined 371 988 patients with acute ischemic stroke who were transported by EMS and enrolled in 1585 hospitals participating in Get With The Guidelines—Stroke from April 1, 2003, through March 31, 2011. Prenotification occurred in 249 197 EMS‐transported patients (67.0%) and varied widely by hospital (range, 0% to 100%). Substantial variations by geographic regions and by state, ranging from 19.7% in Washington, DC, to 93.4% in Montana, also were noted. Patient factors associated with lower use of prenotification included older age, diabetes mellitus, and peripheral vascular disease. Prenotification was less likely for black patients than for white patients (adjusted odds ratio 0.94, 95% confidence interval 0.92–0.97, *P*<0.0001). Hospital factors associated with greater EMS prenotification use were absence of academic affiliation, higher annual volume of tissue plasminogen activator administration, and geographic location outside the Northeast. Temporal improvements in prenotification rates showed a modest general increase, from 58.0% in 2003 to 67.3% in 2011 (*P* temporal trend <0.0001).

**Conclusions—:**

EMS hospital prenotification is guideline recommended, yet among patients transported to Get With The Guidelines—Stroke hospitals it is not provided for 1 in 3 EMS‐arriving patients with acute ischemic stroke and varies substantially by hospital, state, and region. These results support the need for enhanced implementation of stroke systems of care. **(*J Am Heart Assoc*. 2012;1:e002345 doi: 10.1161/JAHA.112.002345.)**

## Introduction

Stroke remains a leading cause of mortality and morbidity in the United States.^1^ Stroke outcomes can be improved significantly with the use of intravenous tissue plasminogen activator (tPA) for acute ischemic stroke.^2–4^ Because the benefits of tPA correlate inversely with the time to therapy administration,^2–5^ the American Heart Association / American Stroke Association (AHA/ASA) have set guidelines for rapid tPA use. A recommended strategy to improve timeliness of stroke evaluation and treatment is for patients with acute stroke to be transported by emergency medical services (EMS) and for EMS to prenotify the hospital of an incoming patient.^6–12^ This prenotification can facilitate earlier mobilization of the stroke team, with activation of stroke processes of care at the receiving hospital even before patient arrival. The AHA/ASA and the National Association of Emergency Medical Services Physicians support the practice of EMS prenotification in stroke care,^6,7^ recognizing its potential to increase the timeliness with which eligible patients receive tPA. Despite these national recommendations, contemporary data are scarce with regard to national use of EMS prenotification and factors associated with its use in a broad cohort of patients with stroke throughout the United States.

The aim of this study was to use data from the AHA/ASA's Get With The Guidelines (GWTG)—Stroke registry to (1) describe the rate of EMS prenotification use; (2) identify variation in EMS prenotification by hospital, state, and region; (3) determine patient and hospital factors associated with EMS prenotification from 2003 to 2011; and (4) evaluate temporal trends in EMS prenotification from 2003 to 2011.

## Methods

GWTG‐Stroke is an initiative undertaken by the AHA/ASA to improve the quality of care delivered to patients with stroke and transient ischemic attack (TIA) through changes implemented at the hospital level. Details of the design and conduct of the GWTG‐Stroke program have been described previously.^5,13^ GWTG uses a Web‐based patient management tool (Outcome Sciences, Inc, Cambridge, MA) to collect clinical data on consecutively admitted patients, provide decision support, and enable real‐time online reporting features. After a year‐long pilot phase, the GWTG‐Stroke program was made available to all hospitals across the United States in April 2003.^5,13^ Each participating hospital received either human research approval to enroll cases without individual patient consent under the common rule or a waiver of authorization and exemption from subsequent review by the hospital's institutional review board. Outcome Sciences, Inc, serves as the data collection and coordination center for GWTG. The Duke Clinical Research Institute serves as the data analysis center and has an agreement to analyze the aggregate, deidentified data for research purposes.

Hospital site personnel were trained to collect data on consecutive patients admitted with the principal clinical diagnosis of acute stroke or TIA by prospective clinical identification, retrospective identification through discharge codes, or a combination of the two. Methods used for the prospective clinical identification of cases involved regular review of several data sources, including emergency department admission logs, ward census logs, intensive care unit logs, and neurology service consultations. Methods used for the retrospective clinical identification of cases included regular surveillance of discharge codes, specifically *International Classification of Diseases, 9th Revision* codes 433.xx, 434.xx, and 436 for ischemic stroke. The eligibility of all acute stroke admissions was confirmed before chart abstraction.^5,13^

Patient data were abstracted by trained hospital personnel and included demographics; medical history; onset time of stroke symptoms (defined as last time patient was known to be well); mode of arrival; arrival time; in‐hospital diagnostic studies, treatments, and procedures; time of initial imaging study; tPA treatment initiation time; tPA complications; in‐hospital death; and discharge treatments, counseling, and destination. The National Institutes of Health Stroke Scale (NIHSS) was used to index stroke severity, when documented. Data collectors at hospital facilities indicated mode of patient arrival under the following classifications: EMS from home/scene, hospital transfer, private transport / walk in, or not documented. If applicable, they further recorded if there had been EMS prenotification to the hospital for the patient in question. The criterion for identifying prenotification was explicit documentation anywhere in the records that advance notification by EMS had occurred and that this notification had indicated, through the use of the word *stroke* or any documentation of signs and symptoms consistent with stroke, that the patient was a suspected stroke patient. All patient data were deidentified before submission. Data on hospital‐level characteristics (eg, number of beds, geographic region, teaching status) were accessed from the American Hospital Association.^14^

A total of 936 702 patients with acute ischemic stroke from 1635 hospitals were enrolled between April 1, 2003, and April 2, 2011. Patients who had in‐hospital strokes (n=15 985) and those transferred in from other acute care facilities (n=94 061) were excluded for the purpose of this study. Of the remaining patients, 404 031 did not arrive by EMS from home/scene. Another 50 637 (12.0%) patients who were transported by EMS had missing prenotification data and were therefore excluded, leaving 371 988 EMS‐transported patients, who constituted the study population. The characteristics of patients with and without documentation of EMS prenotification are shown in online‐only Data Supplement Table I.

### Statistical Analysis

Sociodemographic factors, clinical variables, hospital characteristics, and quality‐of‐care measures were compared between patients transported by EMS with prenotification versus those without prenotification. The Pearson χ^2^ test and Wilcoxon rank‐sum test were used to compare categorical and continuous variables, respectively. Categorical and continuous variables were reported in percentages and median (25th and 75th percentiles) as appropriate. The contemporary pattern in EMS prenotification over calendar years was explored, and the trend from years 2003 to 2011 was tested by using a logistic model including the linear and quadratic terms of calendar year. As additional hospitals joined GWTG‐Stroke over the course of the study, the temporal trend analyses also were performed among the hospitals participating in GWTG‐Stroke in 2003 and 2004 and continuing participation during the entire study period (hospital n=137). Potential variables that might have affected EMS prenotification were identified in the univariate analyses. Candidate patient variables included age, sex, race/ethnicity (white, black, Hispanic, Asian, and other), medical history (including atrial fibrillation or flutter, coronary artery disease or prior myocardial infarction, carotid stenosis, diabetes mellitus, hyperlipidemia, heart failure, hypertension, smoking status, prior stroke or TIA, peripheral vascular disease, and prosthetic heart valve), insurance status (Medicare, Medicaid, no insurance, or other, including health maintenance organization, Veterans Health Administration benefits, or private insurance), and arrival during regular working hours (defined as Monday through Friday between 7 am and 6 pm). Hospital variables that might have affected EMS prenotification included number of beds, geographic region (Midwest, Northeast, West, South), teaching status, average number of patients with ischemic stroke/TIA treated annually, and average number of patients treated with tPA annually. A multivariable logistic regression analysis, with a generalized estimating equations approach to adjust for within‐hospital clustering, was performed to determine factors significantly associated with EMS prenotification. Interactional testing was performed to examine whether the relationship of EMS prenotification and race was modified by different geographic regions. Time trends also were tested in a generalized estimating equations model that adjusted for patient and hospital characteristics. Because the degree of stroke severity is a potential factor affecting both EMS prenotification and patient outcomes, further sensitivity analyses were done on the subset of patients with complete NIHSS data available. These analyses also were performed in the subset of patients arriving within 4.5 hours.

All tests were 2 tailed, with *P*<0.05 considered the level of statistical significance. All statistical analyses were performed in SAS version 9.2 software (SAS Institute, Cary, NC). The authors had full access to the data and take responsibility for its integrity. All authors have read and agree to the manuscript as written.

## Results

Our analysis included 371 988 patients with acute ischemic stroke from 1585 participating sites who arrived by EMS between 2003 and 2011. A median of 131 patients with acute ischemic stroke (25th–75th percentiles: 32–339) were enrolled per hospital. During this 8‐year study period, hospitals reported prenotification for 249 197 patients arriving by EMS (67.0%). If limited to the 1395 hospitals with ≥10 patients entered during the study, the mean rate of EMS prenotification was 62.1%; if limited to the 1089 hospitals with ≥50 patients entered during the study, the mean rate of EMS prenotification was 64.1%. When the analysis was confined to EMS‐transported patients who were potential candidates for tPA treatment under current expanded treatment guidelines (ie, arriving within 4.5 hours from symptom onset, n=135 308), prenotification occurred in 97 511 patients (72.1%). If limited to EMS‐transported patients arriving within 2 hours of symptom onset (n=99 145), prenotification occurred in 73.0%.

Comparisons of patient‐ and hospital‐level characteristics for patients with EMS prenotification versus patients without prenotification are provided in Table 1. Among acute ischemic stroke cases with EMS prenotification, patients were more likely to be younger, white, and male and to have a history of atrial fibrillation. EMS prenotification also occurred more frequently for patients with higher NIHSS scores and for those arriving during facility off‐hours. EMS prenotification was less likely for patients with a history of previous stroke/TIA, diabetes mellitus, peripheral vascular disease, hypertension, dyslipidemia, or heart failure. Time from symptom onset to hospital arrival was shorter in patients with prenotification. Patients with EMS prenotification were more likely to be transported to hospitals that were nonacademic, had larger annual volumes of tPA administration, and were located outside the Northeast.

**Table 1.** Baseline Patient and Hospital Characteristics by EMS Prenotification Status

	EMS Prenotification (n=249 197)	No EMS Prenotification (n=122 791)	*P*
Age, median (25th–75th percentiles), y	76 (64–84)	76 (64–84)	<0.0001
Male, %	46.6	45.7	<0.0001
Race/ethnicity, %			
White, non‐Hispanic	74.6	70.2	<0.0001
Black	13.8	18.1	
Hispanic	5.6	5.4	
Asian	2.3	2.2	
Other	3.7	4.1	
Insurance status, %			
Health maintenance organization/private	26.1	26.7	<0.0001
Medicare	55.2	54.4	
Medicaid/military/Veterans Health Administration	5.7	6.7	
Self/none	3.6	3.8	
Arrival off‐hours, %*	52.5	52.1	0.0077
Time from symptom onset to arrival, median (25th‐75th percentiles), min	113 (55–340)	150 (60–445)	<0.0001
NIHSS, median (25th–75th percentiles)	7 (3–15)	6 (3–13)	<0.0001
NIHSS, %			
>25	2.9	2.1	<0.0001
21–25	4.0	2.8	
16–20	6.1	4.4	
11–15	7.8	5.9	
6–10	12.2	10.0	
0–5	23.4	22.5	
Not documented	43.5	52.4	
Medical history, %			
Atrial fibrillation/flutter	23.4	22.3	<0.0001
Prosthetic heart valve	1.6	1.6	0.61
Previous stroke/TIA	34.3	35.1	<0.0001
Coronary artery disease / prior myocardial infarction	30.5	30.0	0.0028
Carotid stenosis	4.3	4.4	0.31
Diabetes mellitus	31.3	33.3	<0.0001
Peripheral vascular disease	5.1	5.7	<0.0001
Hypertension	80.7	81.8	<0.0001
Smoker	18.1	18.0	0.95
Dyslipidemia	39.6	40.4	<0.0001
Heart failure	6.0	6.6	<0.0001
Medications before admission, %			
Antihypertensive	70.5	71.1	<0.0001
Cholesterol reducer	37.9	38.2	0.18
Diabetic medication	23.2	24.6	<0.0001
Hospital size in beds, median (25th–75th percentiles), n	362 (260–546)	365 (250–524)	<0.0001
Hospital type, % academic	53.4	60.9	<0.0001
Annual volume of ischemic stroke/TIA admissions, %			
≥301	45.7	42.8	<0.0001
101–300	45.6	46.0	
0–100	8.7	11.1	
Annual volume of tPA administration, %			
≥11	23.2	19.4	<0.0001
7–10	35.2	32.8	
0–6	41.6	47.9	
Hospital region, %			
West	20.9	12.6	<0.0001
South	35.3	34.7	
Midwest	19.3	15.3	
Northeast	24.5	37.5	

A median of 131 patients with acute ischemic stroke (25th–75th percentiles: 32–339) were enrolled per hospital.

* Arrival at the hospital that did not occur during Monday through Friday, 7:00 am to 6:00 pm.

NIHSS values were recorded in 199 154 patients. Sex was missing in 0.08%, race/ethnicity in 0.06%, medical history in 6.5%, teaching status 4.4%, and number of hospital beds in 6.5%.

Patient‐ and hospital‐level characteristics that were significantly associated with EMS prenotification are shown in Table. Patient characteristics associated with increased odds of EMS prenotification were younger age; white race; past medical history of atrial fibrillation; and no medical history of previous stroke/TIA, diabetes mellitus, or peripheral vascular disease. In particular, black patients had decreased odds of EMS prenotification when compared to their white counterparts, with an adjusted odds ratio of 0.94 (95% confidence interval 0.92–0.97, *P*<0.0001). Hospital characteristics associated with decreased odds of EMS prenotification were academic affiliation, Northeast location, and smaller annual tPA volumes. When the analysis was confined to patients arriving within 4.5 hours and patients with stroke severity documented with adjustment for NIHSS, the findings were, with few exceptions, similar (Table and online‐only Data Supplement Table II). Greater stroke severity, as indexed by NIHSS, was significantly associated with increased odds of EMS prenotification.

**Table 2.** Patient‐ and Hospital‐Level Characteristics Associated With EMS Prenotification

	Adjusted Ratio	95% Confidence Interval	*P*
Age (per 10 y)	0.98	0.98–0.99	0.002
Female (vs male)	0.99	0.98–1.00	0.03
Race/ethnicity (reference non‐Hispanic white)			
Black	0.94	0.92–0.97	<0.0001
Hispanic	1.00	0.98–1.03	0.81
Asian	0.97	0.93–1.01	0.11
Other	0.89	0.83–0.96	0.003
Medical history			
Atrial fibrillation/flutter	1.05	1.03–1.06	<0.0001
Previous stroke/TIA	0.98	0.96–0.99	0.0007
Diabetes mellitus	0.96	0.95–0.97	<0.0001
Peripheral vascular disease	0.94	0.92–0.97	<0.0001
Annual tPA volume (reference ≥21 patients/y)			
0–10	0.77	0.61–0.96	0.02
11–20	1.12	0.85–1.47	0.43
Academic center	0.65	0.56–0.75	<0.0001
Region (reference Northeast)			
West	2.04	1.63–2.55	<0.0001
South	1.29	1.07–1.55	0.007
Midwest	1.70	1.39–2.07	<0.0001

**Table 3.** Patient‐ and Hospital‐Level Characteristics Associated With EMS Prenotification for Patients Arriving Within 4.5 Hours With Complete NIHSS Data (N=90 135)

	Adjusted Odds Ratio	95% Confidence Interval	*P*
Age (per 10 y)	0.99	0.98–0.99	0.005
Female (vs male)	1.00	0.97–1.02	0.67
Race/ethnicity (reference non‐Hispanic white)			
Black	0.89	0.85–0.93	<0.0001
Hispanic	1.00	0.95–1.05	0.95
Asian	0.90	0.83–0.97	0.008
Other	0.90	0.81–0.99	0.03
Medical history			
Atrial fibrillation/flutter	1.00	0.97–1.03	0.88
Previous stroke/TIA	0.98	0.95–1.01	0.12
Diabetes mellitus	0.98	0.96–1.01	0.20
Peripheral vascular disease	0.94	0.89–0.99	0.03
Annual tPA volume (reference ≥21 patients/y)			
0–10	0.83	0.65–1.06	0.14
11–20	1.15	0.86–1.55	0.34
NIHSS (per 5 units)	1.05	1.04–1.06	<0.001
Academic center	0.69	0.59–0.82	<0.001
Region (reference Northeast)			
West	2.18	1.68–2.84	<0.0001
South	1.31	1.07–1.61	0.01
Midwest	1.52	1.22–1.89	0.0002

There was substantial variation by hospital in the use of EMS prenotification, ranging from 0% to 100%, among hospitals with ≥10 patients (prenotification median 70%; 25th–75th percentiles: 34.0%–92.9%). There was also substantial variation in use of EMS prenotification by state (Figure), ranging from a low of 19.7% in Washington, DC, to a high of 93.4% in Montana (hospital‐level analyses). The numbers of patients and hospitals from each state, along with patient‐level rates by state, are shown in online‐only Data Supplement Table III.

**Figure 1.**
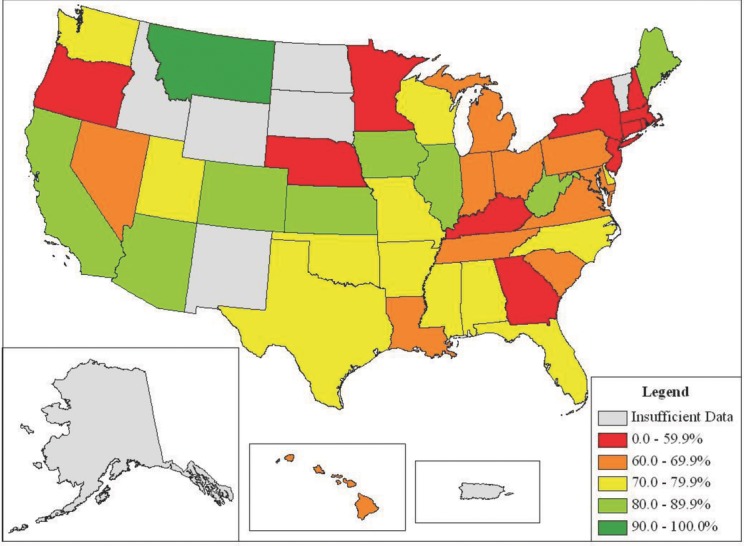
Comparison of EMS prenotification rates by state at the hospital level. States with <4 participating hospitals were excluded.

EMS prenotification rates among patients transported by EMS showed a modest and statistically significant increase, from 58.0% in 2003 to 60.7% in 2004, 66.1% in 2005, 67.0% in 2006, 68.3% in 2007, and 71.1% in 2008, followed by a decline to 65.4% in 2009, 65.5% in 2010, and 67.3% as of April 2011 (*P* temporal trend <0.0001) (Figure). When the analyses were confined to the 137 hospitals continuously participating in GWTG‐Stroke, similar temporal trends were observed (online‐only Data Supplement Figure I). Generalized estimating equations models adjusting for patient and hospital characteristics also showed significant linear and quadratic time trend overall and for continuously participating hospitals.

**Figure 2.**
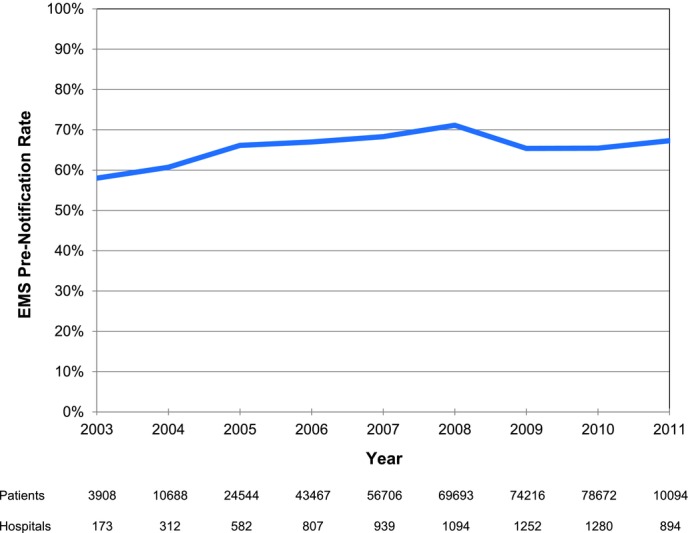
Temporal trends in EMS prenotification, by calendar years, from 2003 to 2011 among patients with acute ischemic stroke transported by EMS. Hospitals N=1585; patients N=371 988. *P* temporal trend <0.0001.

## Discussion

Our analysis of patients with acute ischemic stroke arriving to 1585 hospitals participating in GWTG‐Stroke demonstrates that EMS prenotification is generally underutilized in contemporary practice. Prenotification rates have shown only modest improvements from 2003 to 2011. Furthermore, EMS prenotification practices show striking variation by hospital, ranging from 0% to 100%. Variation also occurred by state and geographic region. Disparities in EMS prenotification use, particularly in regard to patient age, race/ethnicity, and certain comorbid conditions, have been identified in this study and are important to address. Hospital factors, including size, geographic location, and annual ischemic stroke and annual tPA volumes, also were associated significantly with EMS prenotification. These findings demonstrate gaps in the quality of stroke care provided and support the need for initiatives targeted to improve EMS prenotification rates on a national level.

The challenges in meeting the door‐to‐needle performance metric are recognized and well studied.^1^ In a situation in which every minute counts, hospitals and policy makers alike are continuously reevaluating current processes of stroke care in the hope of uncovering methods to improve the timeliness of thrombolytic treatment administration. Prior studies and unpublished data from our analysis propose EMS prenotification as an important strategy for addressing this concern.^8–12,15^ Results from these studies suggest that prenotification practices are associated with more timely evaluation and treatment of patients with acute ischemic stroke. EMS prenotification has been found to facilitate faster imaging and imaging interpretation times, to increase patient eligibility for and administration of tPA, and to increase rates of tPA administered within 60 minutes of arrival.

Among GWTG‐Stroke hospitals in the United States, one third of all EMS‐transported patients do not receive the benefits of prenotification. This highlights a specific area for improvement in stroke treatment practices. A previous study of 118 patients arriving by EMS to the emergency department within 6 hours of symptom onset reported an EMS prenotification rate of 37% and identified EMS prenotification as an underutilized practice.^2008^ Although our analysis reflects a more encouraging national EMS prenotification rate, we found widespread variation in prenotification rates at the hospital, state, and regional levels. Although this range demonstrates that some institutions might require more targeted improvement efforts than others in this regard, it also suggests that very high EMS prenotification rates are feasible and are a reasonable goal. Further investigation into these differential rates could better inform both hospitals and EMS personnel on practices that allow for higher prenotification rates.

Our study further evaluated patient‐ and hospital‐level disparities in EMS prenotification. Age, race/ethnicity, history of atrial fibrillation, and history of diabetes were identified as patient determinants, whereas size, geographic location, and annual volume of patients administered tPA were noted to be significant hospital factors. Older patients and black patients were less likely to have EMS prenotification. EMS were also less likely to prenotify academic hospital facilities and sites that typically have smaller annual tPA volumes. Regionally, the practice of EMS prenotification is least established in the Northeast. Such patient‐level disparities have been documented previously in other settings, both in regard to stroke patient management and more broadly in other areas of health care. Inequalities in the use and timeliness of reperfusion therapies for older, minority, female patients with acute ST‐elevation myocardial infarction have been described.^16,17^ In terms of stroke care, older age and black ethnicity have been associated with decreased odds of door‐to‐needle times ≤60 minutes for acute ischemic stroke.^1^ A recent review by ^Cruz‐Flores et al2011^ highlighted the differential burden of disease and disparities in quality of care delivered to minority patients with stroke. They found that in comparison to white counterparts, minority patients have higher incidence and prevalence of stroke. Black patients, in particular, have a markedly higher burden of disease, with higher rates of functional impairment and death after stroke. Furthermore, minorities tended to have poorer access to acute stroke care, stroke rehabilitation, and stroke prevention services. Other studies have shown that even when minorities have access to medical care, disparities exist in the quality of stroke care received. Blacks were less likely to undergo comprehensive evaluation of an acute stroke and were also less likely to receive tPA.^18–21^ Further research focusing on these disparities, including potential strategies to counter them, will be crucial to the advancement of healthcare equality.

These data support the need for targeted initiatives to improve the national rate of EMS prenotification. This begins with increasing awareness of the benefits of prenotification and disparities in its use among EMS personnel and stroke teams at receiving hospitals. EMS personnel should receive adequate training and protocols that help rapidly and accurately identify potential acute stroke patients and participate in a system of care that includes prenotifying hospitals.

Previous analyses have emphasized the importance of defining minimum thresholds for stroke in EMS training, with a focus on the type of data required for activation of hospital‐based stroke teams.^2007^ Implementation of standardized tools for the detection of acute stroke in the ambulatory setting can further streamline the prenotification process.^6,15^

A stroke system‐of‐care process measure reporting the use of EMS prenotification should be considered. Furthermore, hospital‐based stroke teams should be provided information on improved stroke outcomes associated with prenotification. Institutions should be encouraged to work with EMS and implement stroke systems of care in response to prenotification. Protocols should be in place to facilitate earlier activation of the stroke team and preparation of imaging modalities once prenotification is received so that tPA can be promptly initiated when indicated.^2,5,6,11^

### Limitations

The present study has several limitations. First, participation in GWTG‐Stroke is voluntary. Although it includes >1500 US hospitals, the GWTG‐Stroke registry could self‐select institutions with greater interest in quality improvement, and thus better process performance, than non–GWTG‐Stroke centers.^5,13^ Therefore, our analysis might have overestimated the current use of EMS prenotification. Second, as with all registries, the data presented here are dependent on the accuracy and completeness of abstraction from the medical records. Complete documentation might not have occurred for all patients prenotified by EMS. Conversely, specific stroke patient prenotification could have been documented but could have not actually occurred. The assessment of EMS prenotification was dichotomized as “present” or “absent,” and additional information that might have been communicated to hospitals, such as prehospital stroke screening results and type of symptoms, was not captured. We do not have data on how prenotification varied by each EMS agency. We also do not have any data on the type of first responder, the level of training of the rendering prehospital providers, EMS protocols in use, or characteristics of the EMS agencies. Although our analysis identified several patient and hospital characteristics that affect EMS prenotification, there are likely other important influencing factors not captured here, including EMS agency characteristics and the existence of regional stroke systems of care with routing of patients with stroke directly to designated stroke centers. These are important factors to consider, and further research is warranted. Residual measured and unmeasured confounding variables might have influenced some or all of the findings. With our large study population, small differences in the data in absolute terms were still highly statistically significant.

### Conclusions

Despite guideline recommendations and potential benefits, EMS prenotification occurred in only two thirds of patients with acute ischemic stroke transported by EMS to GWTG‐Stroke–participating hospitals, with only a modest improvement over the past 8 years. Use of EMS prenotification also varies widely by hospital, state, and geographic region among patients transported to GWTG‐Stroke hospitals. Disparities in use of EMS prenotification, particularly in regard to age, race/ethnicity, and certain comorbid conditions, have been identified in this study and will be important to address. Hospital factors, including size, geographic location, and annual ischemic stroke and tPA volumes, also were associated significantly with EMS prenotification. These findings demonstrate gaps in the quality of stroke care provided and support the need for targeted initiatives to improve EMS prenotification rates on a national level.

## Supporting Information

Disclaimer: Supplementary materials have been peer‐reviewed but not copyedited.
